# Relationship between the Normative Need for Orthodontic Treatment and Oral Health in Mexican Adolescents Aged 13–15 Years Old

**DOI:** 10.3390/ijerph17218107

**Published:** 2020-11-03

**Authors:** Álvaro Edgar González-Aragón Pineda, Alvaro García Pérez, Raúl Rosales-Ibáñez, Eduardo Stein-Gemora

**Affiliations:** Department of Public Health Research, Faculty of Higher Studies (FES), Iztacala, National Autonomous University of Mexico (UNAM), State of Mexico C.P. 54090, Mexico; alvaro.garcia@unam.mx (A.G.P.); rosales_ibanez@unam.mx (R.R.-I.); stein@comunidad.unam.mx (E.S.-G.)

**Keywords:** oral health, orthodontic treatment need, malocclusion, adolescents

## Abstract

This cross-sectional study aimed to establish a relationship between the Normative Need for Orthodontic Treatment (NNOT) and oral health among Mexican adolescents aged 13–15 years old. A convenience sample of 424 subjects in Mexico City participated in the study. The dependent variable used was NNOT, which was determined via the dental health component (grades 4 and 5) of the Index of Orthodontic Treatment Need (IOTN). The variables for oral health were as follows: caries experience, oral hygiene, self-reported temporomandibular joint pain, and self-reported bruxism. Logistic regression models were fitted to determine the association between NNOT and oral health. The prevalence of NNOT was 66.0% (280/424), and the crowding was the most prevalent occlusal anomaly with 36.1% (*n* = 135). Multivariate models showed that subjects with NNOT were more than twice as likely to present poor hygiene (OR = 2.56; *p* = 0.001) as subjects presenting crowding (>4 mm) (OR = 1.99; *p* = 0.004) and increased overjet (>6 mm) (OR = 1.74; *p* = 0.046). Those schoolchildren who presented anterior guidance were 72% less likely to present NNOT (OR = 0.28; *p* < 0.001). In conclusion, the risk of presenting NNOT in Mexican adolescents is high, with a prevalence of over 50% of which the most prevalent occlusal anomaly was crowding. On the other hand, poor oral hygiene was associated with crowding and increased overjet.

## 1. Introduction

A malocclusion is defined as the malposition of the maxillary and mandibular teeth to such an extent that it impedes the efficiency of the excursive movements of the jaw that are essential for mastication [1]. Deep overbite, midline deviation, excessive overjet, anterior crossbite, crowding, loss of space, and open bite are occlusal anomalies frequently seen in dental clinics [2].

A recent systematic review showed that adolescents with very severe malocclusion were more likely to experience pain in their jaws or mouths, bleeding gums, or food trapped in their teeth [3]. Further, periodontitis, poor plaque control [4], and temporomandibular disorders are common in adolescents with malocclusion [5,6]. Moreover, malocclusion has been associated with worse quality of life in children and adolescents, mainly because of a lack of self-esteem and altered behaviour [7,8].

Orthodontic treatment indexes are required to both acquire descriptive data on the distribution of treatment needs in populations, and to establish treatment priorities [9,10]. In these sense, the most commonly used indexes in epidemiological studies are the Index of Orthodontic Treatment Need (IOTN) and the Dental Aesthetic Index (DAI) [11,12]. The IOTN index comprises two components, with the first relating to dental and functional health (the Dental Health Component or DHC) and the other based on the aesthetic impact caused by malocclusions (Aesthetic Component or AC).

The IOTN-DHC ranks malocclusion in terms of the significance of various occlusal features for an individual’s dental health and aims to identify those individuals who will most likely benefit from orthodontic treatment [9]. Moreover, the DHC includes five grades that enable the subject to be classified under one of the corresponding categories. Categories 4 and 5 enable the identification of subjects with Normative Need for Orthodontic Treatment (NNOT) based on the presence of one or more severe occlusal anomalies [10]. Previous studies used this index to establish NNOT in adolescents [13,14,15].

Malocclusion has a moderate to high prevalence among adolescents, regardless of the clinical malocclusion measures or dental index used [7]. A systematic review estimated the prevalence of malocclusion at between 15.4–67.6% [16]. Studies conducted in Mexico on a population of 14–20-year-old subjects showed that 37% needed orthodontic treatment: 46.7% presented Class II, and 39.1% presented Class III [17].

Malocclusion can also often be considered a risk factor for caries, given that inadequate tooth alignment facilitates the accumulation and complicates the removal of bacterial plaque [4,18]. Various studies have identified an association between malocclusion and dental caries [16], while others have not been able to establish such an association [19].

The evaluation of the need for orthodontic treatment in a community must be accompanied by knowledge of its impact on oral health; therefore, the objective of the present study was to establish a relationship between NNOT and the oral health of Mexican adolescents aged 13 to 15 years old. The hypothesis proposed is that the presence of NNOT is associated with oral health variables, such as a higher level of caries experience, poor oral hygiene, temporomandibular pain, and bruxism.

## 2. Materials and Methods

### 2.1. Study Population

This cross-sectional study was conducted on a convenience sample of adolescents aged 13 to 15 years enrolled in public schools in Mexico City. The area selected comprised 1,185,772 inhabitants (13.2% of the total population of Mexico City) and is classified by the Consejo Nacional de Población (CONAPO or the National Population Council) as being of a middle socioeconomic level. In this population, 41.4% of people aged over 15 years are educated up to primary level (six years of formal education), while 79.2% have access to health services.

The study protocol was approved by the Ethics Committee of the Faculty of Higher Education Iztacala (FES-I) at the National Autonomous University of Mexico under the agreement/ruling number CE/FESI/012019/1276. The parents or guardians who agreed to participate signed an informed consent form, and the adolescents were also asked to give their own consent.

To estimate a 95% confidence interval, the sample size was calculated with a 5% margin of error for the real value, obtaining a sample size of 442 participants under a non-response rate of 15%.

### 2.2. Variables in the Study

NNOT was the dependent variable evaluated in the present study via the IOTN-DHC [9], which has five grades (1–5) and was used to classify the subjects as follows: Grade 1—No treatment needed; Grade 2—Minor anomaly, no treatment needed; Grade 3—Borderline treatment needed; and, Grade 4 and 5—Treatment needed. The present study uses grades 4 and 5 of the IOTN-DHC [10].

The DHC comprises a hierarchical scale calculated via two stages:

1. The dentition is assessed systematically, thus ensuring that all relevant occlusion anomalies are recorded.
2. If two or more occlusal anomalies are found to be of the same DHC grade, the most severe anomaly is scored.

There is an NNOT if any one of the following occlusal anomalies are present:

- Missing teeth: Space closure or impeded eruption—hypodontia requiring pre-restorative orthodontics or orthodontic space closure to obviate the need for a prosthesis, impeded eruption of teeth, and the presence of supernumerary teeth and/or retained deciduous teeth.
- Overjets: An increased overjet greater than 6 mm.
- Crossbite: Anterior and posterior crossbite (>2 mm)—a greater than 2 mm discrepancy between the retruded contact position and intercuspal position.
- Displacement: Crowding (>4 mm)—contact point displacements greater than 4 mm, as caused by deficient space. Open contact points (>4 mm)—open contact point displacements greater than 4 mm.
- Overbites: Open bite (>4 mm)—lateral or anterior open bites greater than 4 mm. Overbite with gingival or palatal trauma—deep overbite with gingival or palatal trauma.

The following variables were included: NNOT (IOTN-DHC<grade 4/IOTN-DHC ≥grade 4); dental caries (DMFT ≥ 1); oral hygiene, measured via the Simplified Oral Hygiene Index [OHI-S (≤3 = good hygiene/>3 = poor hygiene)]; toothbrushing frequency (<2 times a day/≥ 2 times a day); self-reported temporomandibular joint pain (no/yes); self-reported bruxism (no/yes) and anterior guidance (no/yes); age (in years); and sex (male/female).

### 2.3. Data Collection Methods

The oral examinations were undertaken in a classroom with the participant seated in a school chair with the oral cavity illuminated using artificial light from a portable lamp. A PCP11 probe (Hu-Friedy, Chicago, IL, EE. UU.), a dental mirror (#5), and gauze were used for each participant.

The presence of NNOT was assessed under the operational definition of the IOTN-DHC [10]. Dental caries were evaluated in permanent dentition using the World Health Organization (WHO) criteria based on the decayed (D), missing (M), filled (F) teeth (DMFT) score [20]. Debris and calculus were examined and assessed, with vestibular and palatal/lingual surfaces clinically rated using the OHI-S [21].

The presence of anterior guidance was determined according to the procedure described by Van’t Spijker when the subject presented incisor and canine protection [22]. Incisor protection was evaluated by clinically examining incisors that disengaged the posterior teeth during excursive movements of the mandible. Canine protection was determined by evaluating canines that disengaged the posterior teeth during excursive movements of the mandible.

The data for toothbrushing frequency, temporomandibular joint pain, and bruxism was obtained via the application of a standardized self-reported questionnaire, which comprised the following questions: How often do you brush your teeth? (times a day); Have you ever experienced pain in your jaw joints or difficulty in opening and closing your mouth? (no/yes); and Would you say that you clench or grind your teeth? (no/yes).

### 2.4. Measurement Reproducibility

The measurements were done by an examiner (A.E.G.-A.-P.) that carried out a pre-study calibration or theoretical training by gold standard evaluators (expert dentists) for IOTN-DHC, anterior guidance, DMFT, and OHI-S. Standardizing to IOTN-DHC and anterior guidance were carried out by an orthodontist expert (Filiberto Hernández-Sánchez DDS, MSc; Facultad de Odontología, Universidad Nacional Autónoma de México, Mexico City), obtaining kappa coefficients of 0.91, and 1.00, respectively. On the other hand, the standardization to DMFT and OHI-S was done by the co-author of this study (A.G.-P.), obtaining kappa coefficients of 0.84, and 0.89, respectively.

### 2.5. Statistical Analysis

The data were analyzed using Stata v. 14 (Stata Corp, College Station, TX, USA), while both the total NNOT prevalence and the prevalence by type of occlusal anomalies were calculated. The summary measures, frequencies, and percentages for the categorical data (sex, oral hygiene, toothbrushing frequency, self-reported temporomandibular joint pain, self-reported bruxism, and anterior guidance) and measures of central tendency and dispersion for the quantitative variables (age and caries experience) were then calculated. Bivariate analysis was conducted between the NNOT and oral health variables, while a chi-square test was used for the categorical variables and a Wilcoxon rank-sum test was used on the quantitative variables because they were not distributed normally (Shapiro–Wilk test, *p* < 0.001). Logistic regression models were fitted to determine; (1) the association between NNOT and oral health variables; and (2) the association between oral health and the types of occlusal anomalies. Model diagnostic tests were conducted using the Hosmer–Lemeshow goodness of fit test and statistical analysis of extreme values, and possible interactions were finally analyzed, with values of *p* ≤ 0.05 considered statistically significant.

## 3. Results

A total of 442 subjects were identified, 439 decided to participate, with a non-response rate of 0.7%, while 15 adolescents were excluded because they had orthodontic appliances or had received orthodontic treatments prior to the study, meaning that 424 schoolchildren were included in the present study.

Of the 424 schoolchildren participating in the present study, the mean age was 13.7 (±0.44) years, and 53.1% were female, while no age differences were found by sex (*p* = 0.779). Seventy-two percent (305/424) of the participants had at least one parent with six years of formal education finished, with only 7.8% (33/424) of parents having completed a bachelor’s degree. A total of 24.3% (103/424) had visited the dentist in the last 12 months.

### 3.1. Prevalence of NNOT

The NNOT prevalence was 66.0% (280/424); 52.5% (147/280) presented one anomaly, 35.0% (98/280) presented two anomalies, and 12.5% (35/280) presented three or more (IOTN-DHC ≥ grade 4). Statistically significant differences were not observed among the prevalence of NNOT by age or sex (*p* > 0.05).

The most prevalent occlusal anomaly was crowding, with 36.1% (*n* = 153), while open bite was the least prevalent, with 1.7% (*n* = 7). The prevalence of overbite was higher among males than females (15.1% vs. 8.4%; *p* = 0.033, respectively). The prevalence differences for the remaining occlusal anomalies were not distinct in terms of the sex and age of the participants (*p* > 0.05). Table 1 presents the prevalence of the occlusal anomalies by the sex of the population studied.

### 3.2. Association between NNOT and Oral Health

The present study found that 84.7% (349/424) of participants presented a toothbrushing frequency of twice per day or higher. The prevalence of caries (DMFT ≥ 1) was 89.2% (378/424), with an average of 6.1 (±4.24), while 25.0% (106/424) of the participants presented poor hygiene. Statistically significant differences were not found between these oral health variables and the presence of NNOT.

The occurrence of NNOT was higher in those subjects with poor oral hygiene than in those with good oral hygiene (30.4% vs. 14.6%; *p* < 0.001). The presence of anterior guidance was lower in those presenting NNOT than in those determined to be NNOT-free (11.1% vs. 31.2; *p* < 0.001). Table 2 shows the distribution of the oral health indicators by NNOT prevalence.

### 3.3. Multivariable Analyses

The logistic regression model showed an association between the presence of NNOT and poor oral hygiene, with an odds of 2.48 (95% CI, 1.43–4.30; *p* = 0.001), while subjects presenting anterior guidance were 72% less likely to present NNOT (OR = 0.28; 95% CI, 0.17–0.48; *p* < 0.001). The complete model with the crude and adjusted odds ratios is presented in Table 3.

In Table 4, the dependent variable in the logistic regression model was poor oral hygiene, while the independent variable was an occlusal anomaly when controlling for age, sex, and toothbrushing frequency. It was found that the subjects with crowding (>4 mm) were 99% more likely to present poor hygiene (OR = 1.99; 95% CI, 1.24–3.18; *p* = 0.004), which itself was 74% more likely to present in subjects with increased overjet (>6 mm) (OR = 1.74; 95% CI, 1.01–2.99; *p* = 0.046). When the total NNOT prevalence was included, those with NNOT were twice as likely to present poor hygiene than those who were NNOT-free (OR = 2.56; 95% CI, 1.51–4.36; *p* = 0.001).

## 4. Discussion

This paper provides information about the NNOT with the IOTN-DHC among Mexican adolescents. This is the first report in Mexico to use this index and make possible comparisons with other reports from Latin America and other parts of the world. The present study undertook an analysis of the presence of malocclusions definitely requiring orthodontic treatment, as well as its association with oral health. NNOT was measured objectively, taking a cut-off point that classifies subjects with one or more occlusal anomalies in the most severe categories of the IOTN-DHC (grades 4 and 5). The chronological age range of 13–15 was selected to ensure that the subjects had permanent complete dentition [23], thus ensuring the applicability of the IOTN-DHC parameters and enabling the comparison of the data obtained with the data of other studies. The results of this study expand the known information on the relationship between malocclusions with NNOT and oral health in different populations.

Among the weaknesses of this study is its use of a convenience sample, so we cannot generalize these results to the entire adolescent population of Mexico City. Another limitation of the present study is that, as a cross-sectional study, it was unable to determine causal associations. Moreover, some oral health variables, such as the presence of temporomandibular pain and bruxism, were estimated based on the self-reporting of the study subjects. Finally, the evaluation of caries via the DMFT does not enable the measurement of the different associations between cavitated and non-cavitated lesions and NNOT.

The results of the present study show a NNOT prevalence of 66%, in which crowding was the most common occlusal anomaly identified in the study population (36%). The NNOT prevalence found in the present study was higher than that reported in various other countries (IOTN-DHC≥4). A 1–55% NNOT prevalence is reported for countries in Asia [24,25,26,27,28,29], while 17–36% was observed in Europe [30,31,32] and 25–78% in America [13,14,15,33].

The NNOT detected in the present study mainly corresponded to the presence of crowding >4 mm (36%), indicating a higher prevalence in Mexican adolescents than that found by different studies using the same operational definition (crowding > 4 mm) [24,25,26,27].

One possible explanation for this result is the high level of curative dental service use associated with dental pain and caries experience in Mexico [34], which causes a loss of space due to extensive carious processes or even leads to tooth extraction as an indicated treatment.

On the other hand, poor oral hygiene was associated with the presence of NNOT with more than doubled the odds of presenting NNOT (OR = 2.56; *p* = *0.001*). Similarly, the presence of crowding (>4 mm) and increased overjet (>6 mm) were found to increase the odds of presenting poor hygiene (OR = 1.99 and OR = 1.74, respectively). Prior studies have related the presence of crowding to deficient oral hygiene [4], as both the displacement of the contact point and non-aligned dental arch disharmony can lead to the retention of plaque, thus establishing the importance of the timely treatment of malocclusions to maintain favorable oral health [35].

The presence of dental plaque from poor oral hygiene is recognized as a precursor of caries and periodontal disease [36,37]. Therefore, any factor that promotes plaque retention or hinders its removal contributes to the risk of these diseases. Various types of occlusal anomalies have also been associated with the increased presence of dental plaque. [38,39,40].

Increased overjet (>6 mm) was also found to be associated with poor oral hygiene, with similar results revealing an association between increased overjet and the presence of gingivitis [18] and plaque [40], which may be associated with difficulties in maintaining adequate oral hygiene.

The importance of correcting increased overjet (>5 mm) has previously been established: When presenting permanent dentition, this occlusal anomaly increases the odds of presenting traumatic dental injury [41,42]. Inadequate lip coverage was also identified as one of the main risk indicators [43]. The main causes of injury to anterior permanent teeth are falls, traffic accidents, sports, violence, and collisions with other people or inanimate objects [44].

The present study found an association between NNOT and the absence of anterior guidance, which is explained by a lack of harmony between dental arches due to poor tooth position. Although a contemporary orthodontic objective is the compatibility between static and functional occlusion [45,46], little association has been found between either the lateral occlusal scheme or the lack of a functional scheme and patients’ masticatory functions [47] or the signs and symptoms of temporomandibular disorders [48].

While, other studies have reported an association between poor oral hygiene and caries experience [49,50], the present study did not find an association with NNOT *(p* = *0.820)*, despite finding an 89.2% prevalence of caries (DMFT ≥ 1). A possible explanation for failing to find such an association may be that other risk factors for caries in adolescent Mexicans were not evaluated. Research has reported different factors related to the presence of caries and malocclusion, one of the most important of which is the presence of dental crowding, as that the accumulation of plaque and a lack of toothbrushing lead to a greater prevalence of caries [16].

It is also important for future studies to ascertain the protective effect of saliva on hard tissue in the dental arches, both with and without malocclusion, as a high level of saliva retention is possible in an unaligned dental arch [50,51]. Future studies should consider a longitudinal design that can enable both, the monitoring of the relationship between the early detection of malocclusions, and the establishment of interceptive treatments and their effects on improving oral health variables.

## 5. Conclusions

The risk of presenting NNOT in Mexican adolescents aged 13–15 years old is high, with a prevalence of over 50%, of which the most prevalent occlusal anomaly was crowding. On the other hand, poor oral hygiene was associated with crowding and increased overjet. Therefore, the early detection and treatment of adolescent malocclusion could have a positive impact on physical, functional, emotional, and psychosocial well-being, in addition to improvement in overall oral health.

## Figures and Tables

**Table 1:** Prevalence of occlusal anomalies * by the sex of the population studied.

	Total (%)	Men (%)	Women (%)	*p*
Space closure or impeded eruption				
No	340 (80.2)	157 (78.9)	183 (81.3)	
Yes	84 (19.8)	42 (21.1)	42 (18.7)	0.529 **
Increased overjet (>6 mm)				
No	344 (81.1)	158 (79.4)	186 (82.7)	
Yes	80 (18.9)	41 (20.6)	39 (17.3)	0.390 **
Anterior crossbite (>2 mm)				
No	391 (92.2)	185 (93.0)	206 (91.6)	
Yes	33 (7.8)	14 (7.0)	19 (8.4)	0.589 **
Posterior crossbite (>2 mm)				
No	392 (92.4)	187 (94.0)	205 (91.1)	
Yes	32 (7.6)	12 (6.0)	20 (8.9)	0.266 **
Crowding (>4 mm)				
No	271 (63.9)	134 (67.3)	137 (60.9)	
Yes	153 (36.1)	65 (32.7)	88 (39.1)	0.168 **
Open contact points (>4 mm)				
No	406 (95.7)	188 (94.5)	218 (96.9)	
Yes	18 (4.3)	11 (5.5)	7 (3.1)	0.237 ^†^
Open bite (>4 mm)				
No	417 (98.3)	197 (99.0)	220 (97.8)	
Yes	7 (1.7)	2 (1.0)	5 (2.2)	0.278 ^†^
Overbite: with gingival or palatal trauma				
No	375 (88.4)	169 (84.9)	206 (91.6)	
Yes	49 (11.6)	30 (15.1)	19 (8.4)	0.033 **
TOTAL	424 (100)	199 (100)	225 (100)	

* Normative Need for Orthodontic Treatment (NNOT): Index of Orthodontic Treatment Need-Dental Health Component (IOTN-DHC) ≥ grade 4, ** Chi-square test, ^†^ Fisher’s exact test.

**Table 2:** Distribution of the oral health indicators by the Normative Need for Orthodontic Treatment (NNOT) prevalence *.

	Total *n* = 424	Without NNOT *n* = 144	With NNOT *n* = 280	*p*
Caries experience *	6.09 ± 4.24 (median = 6)	6.03 ± 4.20 (median = 6)	6.12 ± 4.27 (median = 6)	0.852 ^•^
Oral Hygiene ^†^				
Good hygiene	318 (75.0)	123 (85.4)	195 (69.6)	
Poor hygiene	106 (25.0)	21 (14.6)	85 (30.4)	<0.001 ^∞^
Toothbrushing ^‡^ frequency				
<2 times a day	65 (15.3)	23 (16.0)	42 (15.0)	
≥2 times a day	359 (84.7)	121 (84.0)	238 (85.0)	0.792 ^∞^
Temporomandibular joint pain ^‡^				
No	355 (83.7)	127 (88.2)	228 (81.4)	
Yes	69 (16.3)	17 (11.8)	52 (18.6)	0.074 ^∞^
Bruxism ^‡^				
No	240 (56.7)	83 (58.0)	157 (56.1)	
Yes	183 (43.3)	60 (42.0)	123 (43.9)	0.699 ^∞^
Anterior guidance ^§^				
No	348 (82.1)	99 (68.8)	249 (88.9)	
Yes	76 (17.9)	45 (31.2)	31 (11.1)	<0.001 ^∞^

* Index of Orthodontic Treatment Need-Dental Health Component (IOTN-DHC) ≥ grade 4, ** Decayed-Missing-Filled Teeth (DMFT), ^†^ Poor hygiene: Simplified Oral Hygiene Index (OHI-S) ≥ 3, ^‡^ Answers in questionnaires, ^§^ By clinical examination, • Wilcoxon rank-sum test (Mann–Whitney), ^∞^ Chi-square test.

**Table 3:** Logistic regression model of the association between the Normative Need for Orthodontic Treatment (NNOT) prevalence* and oral health variables.

Variables	Crude OR (95% CI) **	*p*	Adjusted ^†^ OR (95% CI) **	*p*
Age (reference < 14 years-old)	1.03 (0.71–1.49)	0.879	0.99 (0.67–1.45)	0.945
Sex (reference = male)	0.82 (0.55–1.23)	0.346	0.77 (0.50–1.20)	0.250
Caries experience ^‡^ (reference < 1 teeth)	1.00 (0.96–1.05)	0.848	0.99 (0.94–1.05)	0.820
Oral Hygiene ^§^ (reference = good hygiene)	2.55 (1.50–4.33)	0.001	2.48 (1.43–4.30)	0.001
Temporomandibular joint pain ^•^ (reference = no)	1.70 (0.94–3.07)	0.076	1.51 (0.82–2.78)	0.182
Anterior guidance ^∞^ (reference = no)	0.27 (0.16–0.46)	<0.001	0.28 (0.17–0.48)	<0.001

* Index of Orthodontic Treatment Need-Dental Health Component (IOTN-DHC) ≥ grade 4, ** OR = odds ratio, CI = confidence interval, ^†^ Adjusted for age, sex, caries experience, oral hygiene, temporomandibular joint pain, and anterior guidance, ^‡^ Decayed-Missing-Filled Teeth (DMFT), ^§^ Simplified Oral Hygiene Index (OHI-S≤3 = good hygiene/OHI-S>3 = poor hygiene), ^•^ Answers in questionnaires, ^∞^ By clinical examination.

**Table 4:** Logistic regression model for the association between poor oral hygiene * and the types of occlusal anomalies with the Normative Need for Orthodontic Treatment (NNOT) **.

Variables ^†^	Crude OR (95% CI) ^‡^	*p*	Adjusted ^§^ OR (95% CI)	*p*
Space closure or impeded eruption	1.68 (1.00–2.82)	0.050	1.51 (0.88–2.59)	0.136
Increased overjet (>6 mm)	1.84 (1.09–3.10)	0.023	1.74 (1.01–2.99)	0.046
Posterior crossbite (>2 mm)	1.90 (0.90–4.04)	0.094	1.75 (0.79–3.84)	0.164
Crowding (>4 mm)	2.18 (1.39–3.41)	0.001	1.99 (1.24–3.18)	0.004

* reference = good hygiene [Simplified Oral Hygiene Index (OHI-S)≤3]; ** Index of Orthodontic Treatment Need-Dental Health Component (IOTN-DHC) ≥ grade 4; ^†^ reference = no; ^‡^ OR = odds ratio, CI = confidence interval; ^§^ Adjusted for age, sex, and toothbrushing frequency.
